# Effect of sedation with detomidine and butorphanol on pulmonary gas exchange in the horse

**DOI:** 10.1186/1751-0147-51-22

**Published:** 2009-05-07

**Authors:** Görel Nyman, Stina Marntell, Anna Edner, Pia Funkquist, Karin Morgan, Göran Hedenstierna

**Affiliations:** 1 Department of Environment and Health, Faculty of Veterinary Medicine and Animal Science, Swedish University of Agricultural Sciences, Skara, Sweden; 2 Orion Pharma Animal Health, Sollentuna, Sweden; 3 Department of Clinical Sciences, Faculty of Veterinary Medicine and Animal Science, Swedish University of Agricultural Sciences, Uppsala, Sweden; 4 Department of Equine Studies, Faculty of Veterinary Medicine and Animal Science, Swedish University of Agricultural Sciences, Uppsala, Sweden; 5 Department of Medical Sciences, Clinical Physiology, University Hospital, Uppsala, Sweden

## Abstract

**Background:**

Sedation with α_2_-agonists in the horse is reported to be accompanied by impairment of arterial oxygenation. The present study was undertaken to investigate pulmonary gas exchange using the Multiple Inert Gas Elimination Technique (MIGET), during sedation with the α_2_-agonist detomidine alone and in combination with the opioid butorphanol.

**Methods:**

Seven Standardbred trotter horses aged 3–7 years and weighing 380–520 kg, were studied. The protocol consisted of three consecutive measurements; in the unsedated horse, after intravenous administration of detomidine (0.02 mg/kg) and after subsequent butorphanol administration (0.025 mg/kg). Pulmonary function and haemodynamic effects were investigated. The distribution of ventilation-perfusion ratios (V_A_/Q) was estimated with MIGET.

**Results:**

During detomidine sedation, arterial oxygen tension (PaO_2_) decreased (12.8 ± 0.7 to 10.8 ± 1.2 kPa) and arterial carbon dioxide tension (PaCO_2_) increased (5.9 ± 0.3 to 6.1 ± 0.2 kPa) compared to measurements in the unsedated horse. Mismatch between ventilation and perfusion in the lungs was evident, but no increase in intrapulmonary shunt could be detected. Respiratory rate and minute ventilation did not change. Heart rate and cardiac output decreased, while pulmonary and systemic blood pressure and vascular resistance increased. Addition of butorphanol resulted in a significant decrease in ventilation and increase in PaCO_2_. Alveolar-arterial oxygen content difference P(A-a)O_2 _remained impaired after butorphanol administration, the V_A_/Q distribution improved as the decreased ventilation and persistent low blood flow was well matched. Also after subsequent butorphanol no increase in intrapulmonary shunt was evident.

**Conclusion:**

The results of the present study suggest that both pulmonary and cardiovascular factors contribute to the impaired pulmonary gas exchange during detomidine and butorphanol sedation in the horse.

## Background

The possibility of producing potent sedation of horses by alpha-2-adrenoreceptor agonists (α_2_-agonists) is one of the greatest improvements in modern equine practice. The dose-dependent sedation and analgesia produced by the α_2_-agonists is reliable for diagnostic procedures and for treatment of various conditions. The central action of the α_2_-agonist is a presynaptic inhibition of noradrenaline accompanied by a decreased sympathetic tone [1]. Alpha-2-agonists also exert physiological effects by their action on peripheral α_2_-receptors [2]. Besides the well recognised and potent cardiovascular changes, sedation with α_2_-agonists in the horse is reported to be accompanied by impairment of pulmonary gas exchange and arterial oxygenation [3-6]. From the studies reported in the horse to date, it is not possible to separate the relative contributions of pulmonary and cardiovascular alterations to the development of impaired arterial oxygenation.

Horses that are deeply sedated with an α_2_-agonist are not unconscious. A sedated horse must be handled with caution, since it may be aroused by stimulation and can respond with dangerous kicks [7-9]. In a situation in which a painful procedure is planned or local analgesia needs to be placed before surgery on the standing horse, accentuation of both sedation and analgesia can be achieved by adding an opioid to the α_2_-agonist [4,10,11]. Butorphanol, a mixed opioid with agonistic and antagonistic properties, has proven effective in such a combination [3,4,12]. There are limited reports on the respiratory effects of butorphanol alone or in combination with the α_2_-agonist detomidine in horses [5,11], but the effects of the combination on pulmonary gas exchange has not been clarified.

With the multiple inert gas elimination technique, developed by Wagner et al. [13] and modified for use in the standing horse [14], the pulmonary gas exchange and a virtually continuous distribution of ventilation-perfusion ratios can be studied.

The aim of the present investigation was to determine the physiological effects, especially on the pulmonary gas exchange, of sedation with detomidine alone and in combination with butorphanol.

## Methods

### Horses

Seven Standardbred trotters (two mares and five geldings) that were considered healthy on clinical examination were studied. Their mean weight was 457 kg (range 380–520 kg) and mean age 5 years (range 3–7 years). Food and water were withheld for approximately 3 hours prior to the sedation procedure. The local Ethical Committee on Animal Experimental in Uppsala, Sweden approved the experimental procedure.

### Catheterisation

All catheterisations were performed with the horse standing and unsedated, after local analgesia with lidocaine (Xylocain^® ^2%, Astra, Sweden). A catheter was introduced percutaneously into the transversal facial artery (18G, Hydrocath TM arterial catheter, Omeda, UK) for systemic arterial blood pressure measurements and collection of arterial blood. A 100 cm pigtail catheter (Cook Europe A/S, Söborg, Denmark) for injection of ice cold saline during thermodilution measurements was introduced by the same technique into the right jugular vein, advanced to the right ventricle and then retracted into the right atrium under pressure-tracing guidance. A thermodilution catheter (7F, Swan-Ganz, Edwards lab., Santa Ana, CA, USA) was inserted with an introducer kit (8F, Arrow Int. Inc., Reading, PA, USA) into the right jugular vein and advanced into the pulmonary artery for mixed venous blood sampling and measurements of core temperature and pulmonary arterial blood pressure. Once correctly placed, the catheters were locked in position with Luer-lock adapters. Further, two infusion catheters (14G, Intranule, Vygone, France) were placed in the left jugular vein.

### Protocol

Detomidine 0.02 mg/kg (Domosedan^® ^vet., 10 mg/ml, Orion Pharma Animal Health, Sollentuna, Sweden) was given intravenously (IV), followed 20 minutes later by butorphanol 0.025 mg/kg IV (Torbugesic^®^, 10 mg/ml, Fort Dodge Animal Health, Fort Dodge, IA, USA). Sampling of blood and expired gas for measurements of gas concentrations by the multiple inert gas elimination technique (MIGET) were performed in the unsedated standing horse (Unsedated) and started 15 minutes after the detomidine injection (Detomidine) and 15 minutes after the butorphanol injection (Detomidine + Butorphanol). The order of the measurements was the same on each occasion, haemodynamic parameters followed by pulmonary function and gas exchange, and the sampling was completed in 5 minutes.

### Measurements of haemodynamic parameters

Systemic arterial and pulmonary arterial blood pressure (SAP and PAP) were measured by connecting the arterial catheters via fluid-filled lines to calibrated pressure transducers (Baxter Medical AB, Eskilstuna, Sweden) positioned at the level of the scapulo-humeral joint. Blood pressure and electrocardiogram (ECG) were recorded on an ink-jet recorder (Sirecust 730, Siemens-Elema, Solna, Sweden). Heart rate (HR) was recorded from the ECG. Cardiac output (Qt) was determined by the thermodilution technique (Cardiac Output Computer Model 9520A, Edwards lab., Santa Ana, CA, USA). A bolus of 20 ml ice cold 0.9% saline was rapidly injected into the right atrium through the pigtail catheter (injection time 3 sec), and the blood temperature was then measured in the pulmonary artery at the tip of the Swan-Ganz catheter and the cardiac output was computed from the recorded temperature change. The mean of at least three consecutive measurements was used.

### Measurements of pulmonary function and gas exchange

Respiratory rate (RR) was measured by observing the costo-abdominal movements, and expired minute ventilation (V_E_) was measured with a Tissot spirometer, range 0.5–685 l (Collins inc., Braintree, MA, USA) attached to the nose mask. Oxygen uptake (VO_2_) was determined by analysing gas from mixed expired air with a calibrated gas analyser (Servomex, Sussex, UK, integrated into an Oximeter 3200, Isler Bioengineering AG, Switzerland). Volume and gas parameters are measured at body temperature and pressure saturated (BTPS). Arterial (a) and mixed venous (v) blood samples for measurements of oxygen and carbon dioxide tensions (PaO_2_, PvO_2_, PaCO_2_, PvCO_2_) and oxygen saturation of haemoglobin (SaO_2_, SvO_2_) were drawn simultaneously and anaerobically into heparinised syringes and stored on ice until analysed (within 30 minutes) by means of conventional electrode techniques with correction of the *p*50 value (ABL 300 and Hemoxymeter OSM 3, Radiometer, Copenhagen, Denmark). Haemoglobin concentration [Hb] was determined spectrophotometrically (Ultrolab system, 2074 Calculating Absorptiometer LKB Clinicon, Bromma, Sweden).

The distribution of ventilation and perfusion was estimated by the multiple inert gas elimination technique [13,14]. Six gases (sulphur hexafluoride, ethane, cyclopropane, enflurane, diethyl ether and acetone), inert in the sense of being chemically inactive in blood, were dissolved in isotonic Ringer acetate solution (Pharmacia, Stockholm, Sweden) and infused continuously into the jugular vein at 30 ml/min from at least 40 minutes before baseline measurements until the collection of the last samples, 15 minutes after butorphanol injection. Arterial and mixed venous blood samples were drawn and simultaneously mixed expired gas was collected from a heated mixing box connected to a nose mask. Gas concentrations in the blood samples and expirate were measured by the method of Wagner et al. [15], using a gas chromatograph (Hewlett Packard 5890 series II, Atlanta, GA, USA). The arterial/mixed venous and mixed expired/mixed venous concentration ratios of each gas (retention and excretion, respectively) depend on its blood-gas partition coefficient and the V_A_/Q (the ratio of alveolar ventilation, V_A _and cardiac output, Q) of the lung. The retention and excretion were calculated for each gas, and the solubility of each gas in blood was measured in each horse by a two-step procedure [15]. The solubilities were similar to those reported previously [14]. These data were then used for deriving the distribution of ventilation and blood flow in a 50-compartment lung model, with each compartment having a specific alveolar ventilation/blood flow ratio (V_A_/Q ratio) ranging from zero to infinity. Ventilation and blood flow in healthy subjects have a log normal distribution against V_A_/Q ratios. Of the information obtained concerning the V_A_/Q distribution, data are presented for the mean and standard deviation of the blood flow log distribution (Qmean and log SDQ, respectively), shunt (perfusion of lung regions with V_A_/Q < 0.005), and the mean and standard deviation of the ventilation log distribution (Vmean and log SDV, respectively). All subdivisions of blood flow and ventilation are expressed in per cent of cardiac output and expired minute ventilation, respectively. The difference between measured PaO_2 _and PaO_2 _predicted from MIGET-algorithms on the basis of the amount of ventilation-perfusion mismatching and shunt was determined. A higher predicted than measured PaO_2 _may reflect diffusion limitation or extrapulmonary shunt.

### Calculations and statistics

From the measurements obtained the following calculations were made, using standard equations. Stroke volume (SV), systemic vascular resistance (SVR) and pulmonary vascular resistance (PVR) as follows:







Diastolic PAP was used in the formula as a substitute for wedge pressure.

For the following calculations, blood gas values measured at 37°C were used.

Alveolar oxygen partial pressure: PAO_2 _= (P_I_O_2 _- (PaCO_2_/R))

(R = Respiratory exchange ratio = 0.8), where P_I_O_2 _= partial pressure of inspired O_2_.

The alveolar – arterial oxygen tension difference (P(A-a)O_2_) was calculated.

Content of oxygen in arterial (a), mixed venous (v), and end-capillary pulmonary (ć) blood:

CzO_2 _= (Hb concentration × 1.39 × oxygen saturation of Hb) + (PzO_2 _× 0.003), where z = a, v, ć. PćO_2 _≈ P_A_O_2_.

Arterial-mixed venous oxygen content difference (C(a-v)O_2_) = CaO_2 _- CvO_2_.

Oxygen delivery: O_2_-del = CaO_2 _× Qt.

Cardiac output (Qt) was also computed through mass balance from measured VO_2 _and the arterio-venous oxygen (or inert gas) content difference (the Fick principle). The cardiac output measurements presented in Table three are based on thermodilution measurements.

For statistical analysis the Statistica 6.0 software package (Statsoft Inc., Tulsa, OK, USA) was used. The data were analysed in a General Linear Model with repeated measures ANOVA. When the ANOVA indicated a significant difference, Tukey's HSD post hoc test was used to determine at what time point there were significant differences within the protocol from baseline and sedation, unless Mauchley's sphericity test indicated significance. In this instance, a planned comparison was applied to define the contrast at each treatment [16]. A p-value less than 0.05 was considered significant. Results are given as mean values ± SD.

## Results

Data on ventilation and blood gases are presented in Table 1, pulmonary gas exchange based on inert gas data in Table 2 and circulation in Table 3.

**Table 1:** Circulatory data (n = 7)

		**Unsedated**	**Detomidine**	**Detomidine-butorphanol**	**GLM – ANOVA**
**HR**	Beats/min	38 ± 8	23 ± 5*	29 ± 5*	p < 0.001

**Qt**	ml/min × kg	72 ± 14	32 ± 10*	44 ± 6*	p < 0.001

**SV**	ml/kg × beat	2.0 ± 0.7	1.4 ± 0.6	1.5 ± 0.3	NS

**SAP mean**	mmHg	116 ± 15	148 ± 14*	137 ± 14*	p < 0.001

**PAP mean**	mmHg	26 ± 2	34 ± 3*	31 ± 4*	p < 0.001

**SVR**	mmHg/ml/min × kg	1.69 ± 0.49	5.01 ± 1.45*	3.16 ± 0.62 *†	p < 0.001

**PVR**	mmHg/ml/min × kg	0.15 ± 0.06	0.31 ± 0.16*	0.15 ± 0.06†	p = 0.017

**O_2 _del**	ml/min × kg	11.4 ± 2.6	5.1 ± 1.8*	6.5 ± 0.8*	p < 0.001

**C(a-v)O**_2_	ml/100 ml	6.1 ± 0.8	8.5 ± 1.8*	7.3 ± 1.1	p = 0.002

**Hb**	g/l	1.15 ± 1.0	1.18 ± 1.3	1.11 ± 1.2	NS (p = 0.062)

Data presented as mean ± SD for heart rate (HR), cardiac output thermodilution (Qt), stroke volume (SV), mean systemic arterial pressure (SAP mean), mean pulmonary arterial pressure (PAP mean), systemic vascular resistance (SVR), pulmonary vascular resistance (PVR), oxygen delivery (O_2 _del), arterial-mixed venous oxygen content difference (C(a-v)O_2_) and haemoglobin concentration (Hb). Results for General Linear Model-ANOVA (GLM-ANOVA), p value for differences between the treatments, NS = non-significant. Differences between treatments are presented with the abbreviations: * = significantly different from unsedated, † = significantly different from detomidine sedation.

**Table 2:** Ventilation and blood gases (n = 7)

		**Unsedated**	**Detomidine**	**Detomidine-butorphanol**	**GLM – ANOVA**
**RR**	Breaths/min	16 ± 3	12 ± 5	10 ± 1* †	p = 0.032

**V_E_**	ml/min × kg	163 ± 36	157 ± 42	114 ± 24*	p = 0.031

**VT**	ml/kg	8.6 ± 1.8	10.6 ± 3.5	10.1 ± 2.4	NS

**PaCO**_2_	kPa (mmHg)	5.9 ± 0.3 (44.3 ± 2.2)	6.1 ± 0.2* (46.1 ± 1.8)	6.4 ± 0.3* † (47.7 ± 2.1)	p < 0.001

**P(A-a)O**_2_	kPa (mmHg)	0.5 ± 0.4 (4.1 ± 2.8)	2.2 ± 0.7* (16.6 ± 5.4)	2.3 ± 1.3* (17.2 ± 9.7)	p < 0.001

**PaO**_2_	kPa (mmHg)	12.8 ± 0.7 (95.7 ± 4.5)	10.8 ± 1.2* (80.7 ± 8.7)	10.6 ± 1.4* (79.2 ± 10.6)	p < 0.001

**PvO**_2_	kPa (mmHg)	4.3 ± 0.3 (32.5 ± 2.6)	3.5 ± 0.5* (26.0 ± 3.5)	3.6 ± 0.2* (27.0 ± 1.7)	p < 0.001

**VO**_2_	ml/min × kg	3.2 ± 0.5	2.4 ± 0.6	2.9 ± 1.0	NS

Data presented as mean ± SD for respiratory rate (RR), expired minute ventilation (V_E_), tidal volume (VT), arterial carbon dioxide tension (PaCO_2_), alveolar-arterial oxygen tension difference (P(A-a)O_2_), arterial oxygen tension (PaO_2_), mixed venous oxygen tension (PvO_2_) and oxygen uptake (VO_2_). For other explanations see Table 1.

**Table 3:** Ventilation/perfusion relationship (V_A_/Q) data (n = 7)

		**Unsedated**	**Detomidine**	**Detomidine-butorphanol**	**GLM – ANOVA**
Percentage **perfusion **of regions with:	**Shunt**	1.1 ± 0.3	1.3 ± 0.4	1.1 ± 0.4	NS
	
	**Normal V_A_/Q**	98.8 ± 0.4	98.5 ± 0.6	98.8 ± 0.4	NS

Percentage **ventilation **of regions with:	**Normal V_A_/Q**	36.0 ± 5.2	30.8 ± 7.5	35.7 ± 7.1	NS
	
	**High V_A_/Q**	0.3 ± 0.5	2.5 ± 3.8	1.6 ± 2.3	NS
	
	**Dead space**	63.6 ± 5.1	66.5 ± 4.2	61.3 ± 4.4 †	p = 0.047

	Log SDQ	0.37 ± 0.09	0.45 ± 0.11*	0.41 ± 0.09	p = 0.002
	
	Log SDV	0.55 ± 0.32	0.85 ± 0.64	0.80 ± 0.59	NS

	Mean Q	0.79 ± 0.21	1.58 ± 0.32*	0.86 ± 0.18 †	p < 0.001

	Mean V	0.95 ± 0.16	2.8 ± 1.7*	1.2 ± 0.33 †	p = 0.029

Data presented as mean ± SD. Log SDQ = logarithmic standard deviation of blood flow (Q) around Q mean (unit V_A_/Q ratio).); Shunt = V_A_/Q < 0.005; normal V_A_/Q = 0.1 < V_A_/Q < 10. Log SDV = logarithmic standard deviation of ventilation (V) around V mean (unit V_A_/Q ratio); normal V_A_/Q = 0.1 < V_A_/Q < 10; high V_A_/Q = 10 < V_A_/Q < 100; dead space = (inert gas) including apparatus dead space: V_A_/Q > 100.

For other explanations see Table 1.

### Unsedated horse

**Figure 1:**
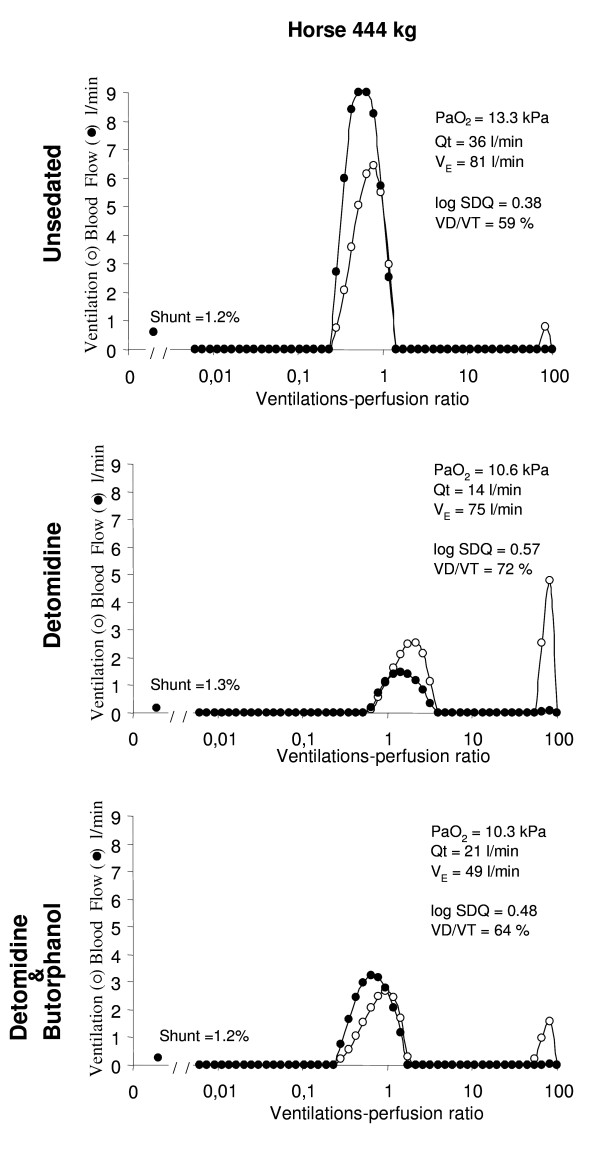
**Distribution of ventilation-perfusion ratio (V_A_/Q) in one horse (444 kg)**. The top panel represent the V_A_/Q distribution in an unsedated horse (Unsedated). The middle panel represent the V_A_/Q distribution 15 minutes after intravenous detomidine administration (Detomidine). The lower panel represent the V_A_/Q distribution 15 minutes after additional intravenous injection of butorphanol (Detomidine & Butorphanol). Note the impaired arterial oxygen tension (PaO_2_) during sedation in the middle and bottom panel. During sedation with detomidine, cardiac output (Qt) decreased and there was an increase in ventilation-perfusion mismatch (broader base of ventilation-perfusion ratio and increased SD of blood flow log distribution (log SDQ)) compared to the unsedated horse. The intrapulmonary shunt was minimal. During sedation with detomidine and butorphanol, the impaired PaO_2_ was a result of persistent low cardiac output and an additional reduction in expired minute ventilation (V_E_). After addition of butorphanol the distribution of V_A_/Q improved as the reduced ventilation and persistent low blood flow matched well. No increase in intrapulmonary shunt was evident during subsequent butorphanol administration.

In the unsedated, standing horse, circulatory data as well as ventilation and pulmonary gas exchange (Tables 1, 2 and 3) were all within normal limits [14]. The distribution of ventilation and perfusion was centered upon a V_A_/Q ratio of approximately 1 (Qmean = 0.79) in all horses (Figure 1, top panel). The overall log SDQ, was 0.37. No regions of low V_A_/Q were noted and in no case was the shunt larger than 1.5% of cardiac output (Figure 1, top panel). The overall log SDV was 0.55, centered around Vmean = 0.95. Bimodal ventilation distribution with an additional mode located within high V_A_/Q ratios (V_A_/Q > 10) was seen in two of seven horses. Dead space (V_A_/Q > 100) (including apparatus dead space, i.e. face mask and non-rebreathing valves of approximately 1 litre) averaged 64%.

### Detomidine sedation

Fifteen minutes after detomidine administration, respiratory rate and expired minute ventilation had not changed significantly, but PaCO_2 _increased slightly but significantly compared to the values in the unsedated horse (Table 1). P(A-a)O_2 _increased and PaO_2 _and PvO_2 _decreased during sedation (Table 1). The shunt remained small but the scatter of V_A_/Q ratio increased as evidenced by a higher log SDQ. The centre of the distribution of ventilation and perfusion increased and Qmean and Vmean were significantly higher than in the unsedated horse (Figure 1, middle panel). Regions with high V_A_/Q ratios were observed in three horses. The predicted PaO_2 _compared to the measured PaO_2 _was slightly but significantly higher compared to values in the standing horse. HR and Qt decreased while increases in vascular resistance and mean SAP and PAP were noted during sedation with detomidine. Second-degree atrio-ventricular (AV) block was recorded during sedation in six of seven horses. VO_2 _did not change, but oxygen delivery decreased significantly and C(a-v)O_2 _was higher during detomidine sedation compared to the values in the unsedated horse.

### Detomidine and butorphanol combination

Addition of butorphanol during the detomidine sedation resulted in a significant decrease in respiratory rate, and a small but significant increase in PaCO_2 _was measured compared to that during detomidine sedation alone (Table 1). Minute ventilation decreased significantly compared to that in the unsedated horse. The cardiovascular changes persisted but the vascular resistance in both the pulmonary and the systemic circulation decreased compared to detomidine sedation alone. Ventilation-perfusion distribution improved and dead space ventilation decreased compared to detomidine sedation. No shunt was seen and the predicted and measured PaO_2 _were similar. Qmean and Vmean did no longer differ from the unsedated horse (Figure 1, bottom panel). The alterations in P(A-a)O_2_, PaO_2 _and PvO_2_, as well as HR, Qt and mean SAP and PAP, that developed during detomidine sedation remained after addition of butorphanol (Tables 1 and 3). Second-degree AV block remained in five of the six horses which showed AV block during detomidine sedation. C(a-v)O_2 _decreased and did not longer differ from the unsedated situation.

## Discussion

It is suggested in the present study that the impaired pulmonary gas exchange during detomidine and butorphanol sedation in the horse originates from both pulmonary and cardiovascular factors. These results are influenced by time and the order of drug administration since the complexity of performing MIGET, including several physiological measurements, limits the frequency of sampling. In the present investigation first MIGET measurements during sedation was taken 15 minutes after detomidine administration and subsequent MIGET measurements during detomidine and butorphanol sedation were taken 35 minutes after detomidine administration. The most pronounced decrease in heart rate during detomidine sedation has been reported between 2–5 minutes after intravenous administration and heart rate remained unchanged between 10 to 30 minutes after injection [8]. In Wagner et al. 1991 [17] detomidine 0.02 mg/kg given intravenously resulted in a significant but stable decrease in cardiac output and respiratory rate compared to unsedated horses between 15 and 60 minutes after administration. In the reported study by Wagner et al. 1991 [17], arterial oxygenation was only significantly decreased at 5 and 15 minutes after sedation. Systemic and pulmonary vascular resistance started to diminish around 30–45 minutes after detomidine injection. The measurements at 15 and 35 minutes after detomidine administration in the present study are thus made at a fairly stable heart rate and cardiac output conditions. The effects on pulmonary gas exchange and oxygenation measured at 35 minuts after sedation is most likely an effect of the additional administration of butorphanol.

### Unsedated horse

The good match between ventilation and perfusion in the standing unsedated horse results in near optimal oxygenation. The narrow distribution of perfusion, with absence of low V_A_/Q regions, negligible intrapulmonary shunt and no diffusion limitation of oxygen, were similar to that found in previous studies [14,18]. The presence of a high V_A_/Q mode, which is usually seen in the resting horse [14], was noted in two of the horses. Interestingly, the horse is able to match ventilation and perfusion as efficiently as young human adults [19,20] and better than sheep [21] despite the fact that the horse has a high vertical lung distance gradient. This shows that the mechanisms for matching ventilation and perfusion are highly efficient in the athletic horse. These mechanisms are probably related to the lung structure and it is proposed that the horse primarily depends for the matching on hypoxic vasoconstriction, i.e. redistribution of blood flow from regions of low ventilation to areas of higher ventilation, by pulmonary vasoconstriction, with only a small contribution from collateral ventilation [22]. Regional PVR is higher in dependent lung regions than in upper ones in the standing horse [23] and this may contribute to the good V_A_/Q match.

### Detomidine sedation

The impaired pulmonary gas exchange and arterial oxygenation during detomidine sedation in the present study reconfirm previous observations during sedation of horses with α_2_-agonists [3,17,24]. Although the reportedly classic causes of an increased P(A-a)O_2_, namely ventilation-perfusion mismatch, failure of alveolar-end capillary diffusion equilibration and right-to-left vascular shunt, have been proposed as presumable mechanisms, extrapulmonary contributors, e.g. extrapulmonary shunt and cardiac output alterations, are possible [17].

It has been reported that the physiological changes induced by α_2_-agonist may be dose-dependent [17,25]. Also, since the physiological effects induced by α_2_-agonists are transient, the choice of methodology and time points for data sampling probably affect the results. The detomidine dose of 0.02 mg/kg used in the present study is a clinically effective sedative dose in most horses [8]. The measurements of cardiovascular and pulmonary function were performed at 15 minutes after intravenous injection of the detomidine. The significant increase in P(A-a)O_2 _was mainly attributed to increased V_A_/Q mismatch as a reduction of cardiac output.

The cardiac output was reduced by 56% which is in line with the literature [17,26]. Since, the cardiac output measurement may be inaccurate during bradycardia with AV block, cardiac output was both measured by thermodilution and calculated according to the Fick principle. The results were in good agreement. In the present study no increase in either pulmonary shunt or low V_A_/Q was evident in the horses (Figure 1). The significantly increased V_A_/Q mismatch (log SDQ) measured during sedation might be caused by a larger vertical difference in perfusion. The shift of the V_A_/Q distribution to a higher range of V_A_/Q ratios during detomidine sedation (Figure 1) was caused by a significant reduction in pulmonary perfusion with unaltered ventilation.

In the healthy human or animal the expected response on increased V_A_/Q mismatch is mitigated by an increase in the overall lung V_A_/Q ratio, thereby increasing the alveolar ventilation and raising both alveolar and arterial PO_2 _[17,26,27]. The absence of ventilatory response to the detomidine-induced hypoxaemia may be due either to decreased ventilatory responsiveness or to decreased receptor sensitivity. However, in the present study, detomidine administration did not result in changes in respiratory rate or minute ventilation. An unaffected respiratory rate is in line with some reports, although others have found a decreased or increased respiratory rate in healthy detomidine-sedated horses [24,28].

Interestingly, Wagner et al. [17] reported that the respiratory rate was significantly reduced 15 minutes after sedation and remained low during the study period of two hours. Also, the slightly increased PaCO_2 _suggested that there was some degree of hypoventilation. The lack of a compensatory increase in alveolar ventilation during sedation with α_2_-agonists means that the arterial blood gases are not corrected. It has been demonstrated that α_2_-adrenergic receptors are present in the carotid body and that such agonists exert an inhibitory influence on the chemoreceptor response to hypoxia [29]. Further, dexmedetomidine administered intravenously to dogs resulted in a diminished response to increased CO_2_, lasting for approximately 2 hours [30]. In agreement with earlier reports on α_2_-agonists [8,17], sedation with α_2_-agonists was associated with a significant increase in pulmonary and systemic arterial blood pressure. Although the distribution of blood flow from hypoxic regions in the lung to ventilated areas is highly efficient in the pony [22], it is possible that the elevated PAP may disturb this mechanism for matching of the perfusion to ventilated areas and thereby also contributes to impaired arterial oxygenation [31].

The slightly higher PaO_2 _predicted by the multiple inert gas elimination technique (MIGET) compared to the measured PaO_2 _may be due to diffusion limitation or extra-pulmonary reasons. Diffusion limitation can be caused by a limited gas equilibration time or by structural changes of the alveolar-capillary interface. Diffusion limitation seems unlikely as the cardiac output was not high enough to cause time limited gas equilibration and no clinical signs of pulmonary oedema were seen. Administration of the α_2_-agonist dexmedetomidine to dogs has been shown to decrease cardiac output with 50%, resulting in decreased perfusion of skin and muscle without decrease in blood flow to the heart [32]. Venous blood from the heart enters the arterial circulation through the Thebesian vein, without going through the lung and is not a part of the MIGET measurements. Thus, the difference between predicted and measured PaO_2 _during detomidine sedation may be due to a proportionally larger contribution from the Thebesian vein to the arterial circulation which lowers the PaO_2_.

A reduction in mixed venous PO_2 _from 4.3 to 3.5 kPa accompanied the decrease in arterial oxygenation during detomidine sedation in the present study. A reduction in cardiac output decreases PvO_2 _when oxygen consumption remains unchanged. Although there was a tendency for increased haemoglobin concentration and oxygen carrying capacity in the blood during detomidine sedation this effect was overridden by the pronounced decrease in cardiac output. The final result was an overall decrease in oxygen delivery to the tissue and increased oxygen extraction. The reduced PvO_2 _further reduces PaO_2 _for the same degree of ventilation-perfusion mismatch [33]. Thus, the slight but significantly increased V_A_/Q mismatch measured during sedation in the present study further aggravated the pulmonary gas exchange, especially in the presence of impaired perfusion.

### Detomidine and butorphanol combination

This drug combination is reported to have minimal effects upon the cardiovascular system [11] and usually does not cause any circulatory changes beyond those induced by the α_2_-agonist alone although there may be a slight further respiratory depression [3,4]. In the present study, the only clear effect on pulmonary gas exchange by the combination of detomidine and butorphanol was a further decrease in ventilation, with additional increase in PaCO_2_. This finding is probably an effect of butorphanol since the effect of the detomidine administered intravenously 35 minutes earlier is most likely diminished [17,28]. Lavoie et al. [5] found that a combination of detomidine and butorphanol in healthy horses as well as in horses with pre-existing respiratory dysfunction affected the respiratory function.

In the present study the increased P(A-a)O_2 _persisted when butorphanol was additionally administered but the contribution of the causative factors changed. After butorphanol administration, the V_A_/Q distribution improved and both Qmean and Vmean were normalised. The shift of V_A_/Q distribution to relatively lower but normal range was achieved by the reduction in ventilation, which now matched the reduced blood flow (Figure 1). Interestingly, the fraction of dead space ventilation was reduced compared to values during sedation with detomidine alone. This possibly reflects an improved distribution of blood flow, since vascular resistance was reduced compared to the values during detomidine sedation. This is in line with earlier investigation on sedation in the horse [17] that has showed a reduction in vascular resistance over time.

## Conclusion

The results of the present study suggest that both pulmonary and cardiovascular factors contribute to the impaired pulmonary gas exchange during detomidine and butorphanol sedation in the horse. A significant reduction in blood flow and increase in V_A_/Q maldistribution are the major contributors to the alveolar-arterial oxygen tension difference during sedation with detomidine. After addition of butorphanol P(A-a)O_2 _remained impaired despite the improved V_A_/Q distribution. This was caused by decreased ventilation, induced by the butophanol administration, which matched a persistent low blood flow. No increase in intrapulmonary shunt compared to unsedated horses was evident during detomidine sedation or subsequent butorphanol administration.

## Competing interests

SM is employed by Orion Pharma Animal Health, Sollentuna, Sweden. This investigation was carried out as a part of Marntell's PhD thesis at the Department of Clinical Sciences, Faculty of Veterinary Medicine and Animal Science, Swedish University of Agricultural Sciences, Uppsala, Sweden.

## Authors' contributions

GN planned and organised the study and was in charge of the practical work. GN and SM collected and analysed data and prepared major parts of the manuscript. GH participated in interpretation of the pulmonary function and in critically revising the manuscript. AE, PF and KM contributed in collection of samples and the laboratory work as well as handling horses. All authors read and approved the final manuscript.
